# Functional Dynamics Inside Nano- or Microscale Bio-Hybrid Systems

**DOI:** 10.3389/fchem.2018.00621

**Published:** 2018-12-18

**Authors:** Zhuojun Dai, Shuqiang Huang

**Affiliations:** Institute for Synthetic Biology, Shenzhen Institutes of Advanced Technology, Chinese Academy of Sciences, Shenzhen, China

**Keywords:** nano/microgels, synthetic biology, fabrication technology, bio-hybrid system, dynamics

## Abstract

Soft nano- or microgels made by natural or synthetic polymers have been investigated intensively because of their board applications. Due to their porosity and biocompatibility, nano- or microgels can be integrated with various biologics to form a bio-hybrid system. They can support living cells as a scaffold; entrap bioactive molecules as a drug carrier or encapsulate microorganisms as a semi-permeable membrane. Especially, researchers have created various modes of functional dynamics into these bio-hybrid systems. From one side, the encapsulating materials can respond to the external stimulus and release the cargo. From the other side, cells can respond to physical, or chemical properties of the matrix and differentiate into a specific cell type. With recent advancements of synthetic biology, cells can be further programed to respond to certain signals, and express therapeutics or other functional proteins for various purposes. Thus, the integration of nano- or microgels and programed cells becomes a potential candidate in applications spanning from biotechnology to new medicines. This brief review will first talk about several nano- or microgels systems fabricated by natural or synthetic polymers, and further discuss their applications when integrated with various types of biologics. In particular, we will concentrate on the dynamics embedded in these bio-hybrid systems, to dissect their designs and sophisticated functions.

## Introduction

Hydrogels are polymeric materials that consist of crosslinked three-dimensional (3D) networks. Due to their porosity and high water content, they can serve as ideal matrices for controlled release of biomolecules, cells encapsulation and delivery, as well as scaffolds in tissue engineering (Minh and Lee, 2010; Naderi et al., 2011; Smeets and Hoare, 2013; Suo et al., 2018). Stimulus-sensitive hydrogels, in particular, can swell or shrink in response to multiple modes of external stimulus such as temperature, pH, ionic strength, and light (Gorelikov et al., 2004; Gu et al., 2013; Trongsatitkul and Budhlall, 2013). Compared with macrogels (millimeters to a few centimeters), nano- or microgels (several nanometers to hundreds of microns) can respond more quickly to environmentally-triggered changes, enabling their uses in controlled and regulated applications (Dai and Ngai, 2013; Smeets and Hoare, 2013; Headen et al., 2014). Additionally, their small size allows administration by various routes (e.g., injection) (Liu and Garcia, 2016). These features provide numerous opportunities for versatile applications.

On the other hand, synthetic biology has emerged as a powerful approach for engineering systems with predicted applications (de Lorenzo, 2008; Ye and Fussenegger, 2014; Din et al., 2016). Advances in programming single cell or cell populations with well-characterized functions have demonstrated their potentials in broad areas spanning from biotechnology to new medicines (Bacchus et al., 2013; Cao et al., 2017; Karig, 2017; Tay et al., 2017; Villarreal et al., 2018). A key focus for synthetic biology is engineering bacteria for medical and environmental related applications (Ye and Fussenegger, 2014; Slomovic et al., 2015; Higashikuni et al., 2017; Karig, 2017). However, these engineered cells need to be trapped and separated from the surrounding tissues and environment to avoid side effects (Chang, 2005; Auslander et al., 2012). Microgels mediated encapsulation provides a solution since the porous structure allows permeability of small molecules, such as oxygen, nutrients, growth factors, and signaling molecules. At the same time, it prevents large molecules, such as immunoglobulins and immune cells (high molecular weight complexes) from reaching the encapsulated cells, and encapsulated cells from reaching tissues and environment (Auslander et al., 2012; Ye and Fussenegger, 2014).

By far, the researchers have successfully integrated the nano- or microgels and biologics to demonstrate a broad range of applications. Nano- or microgels are integrated with therapeutics to treat diabetes and cancer (Das et al., 2006; Sung et al., 2012; Gu et al., 2013; Kanamala et al., 2016), or assembled with stem cells in the tissue engineering (Das et al., 2014; Caldwell et al., 2017). Recently, the integration of nano- or microgels with engineered cells has proven their superiority in diseases detection and treatment (Shao et al., 2017; Xue et al., 2017; Wang et al., 2018). We will start from the basic characteristics, crosslinking strategies, and synthesis scheme of several nano- or microgels systems, and later discuss their applications when integrated with various biologics. In particular, we will focus on the functional dynamics inside the bio-hybrid systems, from either materials or cells side.

## Nano- or Microgels Systems

Both natural and synthetic polymers have been used to synthesize the nano- or microgels (Silva et al., 2004; Oh et al., 2008; Kim et al., 2014). Although the source and batch variations provide limitations, natural polymers are generally bio-compatible, biodegradable, and have been successfully used in clinical applications (Dhandayuthapani et al., 2011). In comparison, nano- or microgels made by synthetic polymers normally have predictable and reproducible physical and chemical properties. The systems made by either natural or synthetic polymers can be further tailored for special purposes, such as sensing and targeting (Gan et al., 2005; Pich and Richtering, 2010). In this section, we will review several well-known and widely used natural and synthetic polymer systems, including their physical and chemical properties, as well as crosslinking strategies.

### Natural Polymer Systems

Natural polymers include proteins (silk, collagen, etc.), polysaccharides (alginate, chitosan, cellulose, etc.), and polynucleotides (DNA and RNA). Especially, two natural polysaccharides: alginate and chitosan, are widely utilized in biomedical and bioengineering fields.

#### Alginate

Alginate is an anionic polysaccharide (α-D-mannuronic acid and β-L-guluronic acid) derived from seaweed, and has been broadly used due to its biocompatibility, flexibility in modifications, and mild crosslinking conditions (Lee and Mooney, 2012; Szekalska et al., 2016). Divalent cations are the most frequently used crosslinking agents to prepare alginate nano- or microgels. The gel structure forms when the divalent cations bind to guluronate blocks of one alginate chain and the other adjacent chain (Lee and Mooney, 2012). Calcium chloride is commonly used as a divalent cation related crosslinking agent. However, its high solubility and fast diffusion in aqueous solution typically lead to rapid but poorly controlled gelation. To control the gelation rate in order to attain a more uniform gel structure and better mechanical integrity, one approach is to use a buffer system containing phosphates. The phosphate groups will compete with carboxyl groups from alginate for calcium ions and slow down the overall gelation rate. Calcium sulfate and calcium carbonate, because of their lower solubility, can also be used to control the gelation rate and derive a more uniform crosslinking structure (Augst et al., 2006; Lee and Mooney, 2012).

The major concern about the ionically crosslinked alginate nano- or microgels is their limitation in long-term stability, since monovalent cations will gradually replace divalent cations and dissociate the network in the physiological condition. Therefore, cationic polymers such as poly (L-lysine) (PLL), have been utilized to coat the surface of alginate nano- or microgels based on their electrostatic interactions (polyanions and polycations) to enhance the overall stability. In another approach, chemical crosslinking (covalent bonding) can be applied parallelly with ionic crosslinking. For example, a photo crosslinked alginate-methacrylate microgel was prepared by integrating the ionic and chemical crosslinking strategies (Li et al., 2017). Ionic crosslinking was used in extrusion process for beads formation due to its fast gelation speed. Photo-crosslinking, which proceeds much slower, was used to increase the stability of beads due to the irreversibility of covalent bonding.

#### Chitosan

The positively charged polysaccharide chitosan is essentially composed of β-(1,4)-linked glucosamine units (2-amino-2-deoxy-β-d-glucopyranose) together with some proportion of N-acetylglucosamine units (2-acetamino-2-deoxy-β-d-glucopyranose), and prepared by partial deacetylation of the natural polymer chitin (Anitha et al., 2014). Chitosan is biocompatible, degradable and has been tested for biomedical applications ranging from wound dressing, drug delivery to tissue engineering (Lopez-Leon et al., 2005; Anitha et al., 2014). It is typically dissolved in acidic media (e.g., acetic acid) due to the existence of amine groups and high crystallinity.

Chitosan can form hydrogels by either chemical or ionic crosslinking. The most frequently used chemical crosslinkers for chitosan are dialdehydes, such as glutaraldehyde (Suh and Matthew, 2000; Berger et al., 2004). The reaction between amine groups (chitosan) and aldehyde groups (dialdehydes) forms covalent imine bonds. Although the reaction can proceed in gentle conditions (aqueous media, room temperature), there are concerns due to the toxicity of dialdehydes (Berger et al., 2004). Alternatively, other chemical crosslinkers, such as genipin, which possesses similar crosslinking properties with glutaraldehyde, but without corrosiveness, cytotoxic and carcinogenic side effects, are preferably developed and applied in recent studies (Skop et al., 2013).

Compared with chemical crosslinking, ionic crosslinking of chitosan is much simpler and milder, and therefore applied widely in medical or pharmaceutical applications. The networks form due to the electrostatic interactions between positively charged amine groups from chitosan backbones and the negatively charged crosslinkers. Both polyanions, such as heparin, or anionic molecules, such as tripolyphosphate (TPP) or phytic acid (PA) can be applied as ionic crosslinkers (Gan et al., 2005; Lee et al., 2011). To reinforce the stability and mechanical property of the chitosan network, the chemical and ionic crosslinking strategies can also be integrated. For example, Skop et al. has demonstrated the fabrication of genipin covalently crosslinked chitosan–heparin complex microspheres (Skop et al., 2013). These microspheres were shown to be a more stable system. Fine tuning in the size, surface charge or other characteristics of nano- or microgels can be obtained by varying compositions and preparation conditions (Gan et al., 2005).

### Synthetic Polymer Systems

Multiple synthetic polymers have been used to construct nano- or microgels. Especially, we discussed two synthetic polymers: poly(N-isopropylacrylamide) and poly (ethylene glycol), due to their stimulus responsiveness and bio-compatibility.

#### poly(N-isopropylacrylamide)

Poly(N-isopropylacrylamide) (pNIPAM) nano- or microgels systems have been one of the most widely studied systems due to its unique thermal sensitivity. Nano- or microgels made of pNIPAM have a lower critical solution temperature (LCST) at approximately 32°C (Wu and Zhou, 1996; Dai and Wu, 2010). The nano- or microgels swell or shrink when temperature is below or above the LCST, with their size changing by more than an order of magnitude (Dai and Wu, 2010). The incorporation of other monomers, such as acrylic acid (AA) or methacrylic acid (MAA) further equips the system with pH and ionic strength responsiveness (Gan et al., 2009; Dai et al., 2015).

pNIPAM nano- or microgels are usually prepared by precipitation polymerization (Acciaro et al., 2011; Li et al., 2012; Dai and Ngai, 2013). The NIPAM monomers, *N, N'*-methylenebis-acrylamide (MBA) crosslinkers, and free radical initiators (e.g., potassium persulfate) are all dissolved in water, and the polymerization is initiated by heating the system to 60–70°C. Besides pNIPMA, various comonomers (such as acrylic acid) can be co-polymerized into the nano- or microgels networks during the synthesis process, and the nano- or microgels size can be controlled from tens of nanometers to microns (Karg and Hellweg, 2009; Dai and Ngai, 2013).

#### Poly (Ethylene Glycol)

As another typical synthetic polymer, poly (ethylene glycol) (PEG) nano- or microgels are also widely studied and used. Especially, its utilization has been approved by FDA (The U.S. Food and Drug Administration) due to its biocompatibility, low protein adhesion, and non-immunogenicity (Chang et al., 2017). The end hydroxyl groups of PEG molecules can be readily modified with organic functional groups, such as carboxyl, thiol and acrylate, which greatly facilitates the nano, or microgels assembly, as well as the bioactive agents conjugation (Chang et al., 2017). PEG nano- or microgels can be fabricated by either batch or continuous fashions. For example, Tan et al. fabricated a PEG-protein nanogels by mixing PEG (modified with benzaldehyde end groups) and protein molecules (lysine residues) in a batch manner (Tan et al., 2012). In another work, Guerzoni et al. used a microfluidic device to generate PEG microgel capsules in a continuous way (Guerzoni et al., 2017). The resultant PEG microgels had a hollow core facilitating the bioactive molecules loading, while the shells were assembled by photo-crosslinking six-armed acrylated star-shaped PEG.

## Nano- or Microgels Fabrication

**Figure 1:**
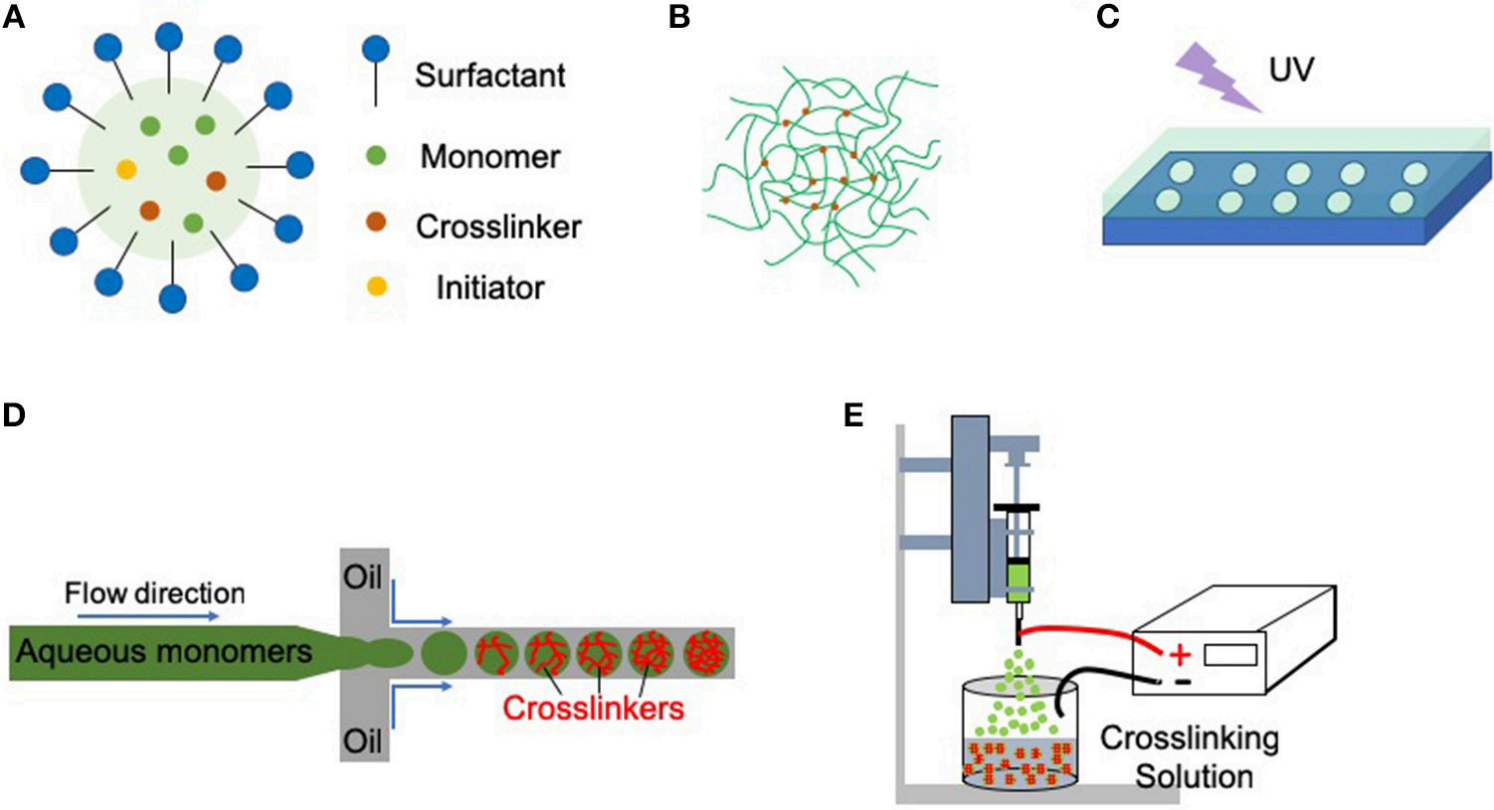
Nano- or microgels can be fabricated by multiple methods. **(A)** Emulsion polymerization (oil-in-water). Droplets of monomers (the oil) are emulsified (with surfactants) in a continuous phase of water. The initiators (often thermally-activated) start the radical polymerization process and form the nano- or microgels. **(B)** Precipitation polymerization. Precipitation polymerization begins initially as a homogeneous system in a continuous phase, where the monomers and initiators are completely soluble. Upon initiation, polymer networks precipitate, and growth of microgel particles proceeds with the absorption of monomers and initiators. No surfactants or stabilizing agents are needed for precipitation polymerization. **(C)** Imprint lithography. Hydrogel precursors are filled into a template that acts as a mold. UV light is then used to photo-polymerize the precursors inside the mold. **(D)** Microfluidic polymerization. Multiple phases (e.g., monomers in the aqueous phase, crosslinkers in the oil phase) meet in a junction geometry (e.g., T-junction) where droplets form. Crosslinking occurs after the formation of these droplets. **(E)** Electrospray. The polymer solution is pushed through a syringe pump. An electrostatic potential is applied between the nozzle or syringe needle and the crosslinking solution. The formed nano- or microdroplets further crosslink in the crosslinking solution.

Various synthesis routes are available to generate nano- or microgels (Figure 1; Liu and Garcia, 2016). Emulsion or precipitation polymerization techniques involving polymerization in bulk solution are the most frequently used methods, especially for synthetic polymers (Saunders and Vincent, 1999; Pich and Richtering, 2010). Lithographic technique utilizes a master template or mask to control the size and morphology of nano- or microgels (Helgeson et al., 2011). Microfluidic polymerization by a microfluidic device is a continuous synthesis route, which offers precise control over nano- or microgels size, morphology, and polydispersity (Wheeldon et al., 2010; Eydelnant et al., 2014). Electrospray technique can also be utilized to fabricate microgels, and the technique is convenient to encapsulate the living cells (Tapia-Hernandez et al., 2015).

### Emulsion Polymerization and Precipitation Polymerization

Emulsion polymerization generally involves an aqueous phase containing surfactants, an oil phase containing monomers and crosslinkers, and initiators to start the radical polymerization process (Figure 1A; Tobita et al., 2000). The system is then homogenized to generate droplets of monomer in oil phase, surrounded by the water phase. The surfactants stabilize the droplets and prevent them from aggregation. By initiating the reaction (e.g., heating the system for a thermal initiator), the initiators react to form free radicals that start the polymerization process. In a variant of this technique (inverse emulsion polymerization), the aqueous phase contains the monomers and crosslinkers while the initiators can be in either of two phases. The particle size can be tuned by adjusting parameters such as the homogenization speed and the reaction temperature (Tobita et al., 2000). Biologics, such as functional proteins or drugs can be loaded by incubation with collected nano- or microgels after synthesis (Liu and Garcia, 2016). The system for emulsion polymerization may also be adjusted to remove the heating step, or the surfactants, to facilitate the biologics loading.

Resembling emulsion polymerization, precipitation polymerization also proceeds in a batch process. However, precipitation polymerization starts in a continuous phase, where the monomers and initiators are completely soluble, and no stabilizing agents are needed (Figure 1B). After initiating the reaction (e.g., increasing the system temperature), a spontaneous nucleation process occurs and polymerization proceeds.

### Lithographic Processes

Lithographic technique fabricates nano- or microgels by templating hydrogels at nano- or micro-scale level. This method needs a template to control both the size and morphology of the product (Helgeson et al., 2011). For example, imprint lithography utilizes a template that acts as a mold for the hydrogel precursors, and UV light is then applied to polymerize the precursors inside the mold (Figure 1C). Nano- or microgels are recovered from the mold, typically by mechanical delamination post-synthesis. Biologics are often loaded by incubation with collected nano- or microgels after polymerization to avoid the UV light exposure. Alternatively, biologics can also be mixed with the hydrogel precursors if the crosslinking condition is mild. Lithographic technique provides a precise control over particle size, morphology, and monodispersity by tuning the template characteristics.

### Microfluidic Polymerization

Microfluidic synthesis technique requires a lithographically fabricated microfluidic device (typically manufactured by polydimethylsiloxane), and generates nano-, or microgels droplets one at a time in a continuous manner. Taking emulsion-based microfluidic system as an example, multiple phases (monomers in aqueous, crosslinkers in oil, etc.) meet in a junction geometry and droplets form. Crosslinking will occur right after the formation of these droplets (Figure 1D). The biologics are often loaded by mixing with the monomers in the aqueous phase. Characteristics of nano- or microgels, including size, morphology, and size distribution can be precisely controlled by several parameters, such as the nozzle diameter and flow rate (Headen et al., 2014).

### Electrospray Fabrication

In the electrospray, the liquid flows from a capillary nozzle through an electric field that disrupts a large droplet into nano- or microdroplets. The nano- or microdroplets are then collected and homogenized in the crosslinking solution (Figure 1E). When an electric field is applied to a droplet, the electric charge generates an electrostatic force into the droplet. Nano- or microdroplets will form when the electrostatic force overcomes the cohesive force of the droplet. The particle size and the size distribution can be tuned by adjusting parameters, such as the flow rate of the solution and the applied electric potential (Tapia-Hernandez et al., 2015). Electrospray has been developed into a mature technology with encapsulation systems (e.g., alginate/Ca^2+^), and is particularly convenient to encapsulate medicines, foods, and microorganisms.

## Dynamics Inside bio-hybrid Systems

Nano- or microgels systems have been widely used in diverse fields. When integrated with drugs, proteins and living cells, these bio-hybrid systems are endowed with various desired functions. Especially, researchers have created multiple modes of functions related dynamics inside these bio-hybrid systems, either from the side of materials or cells.

### Controlled Release Systems—Response From Materials

**Figure 2:**
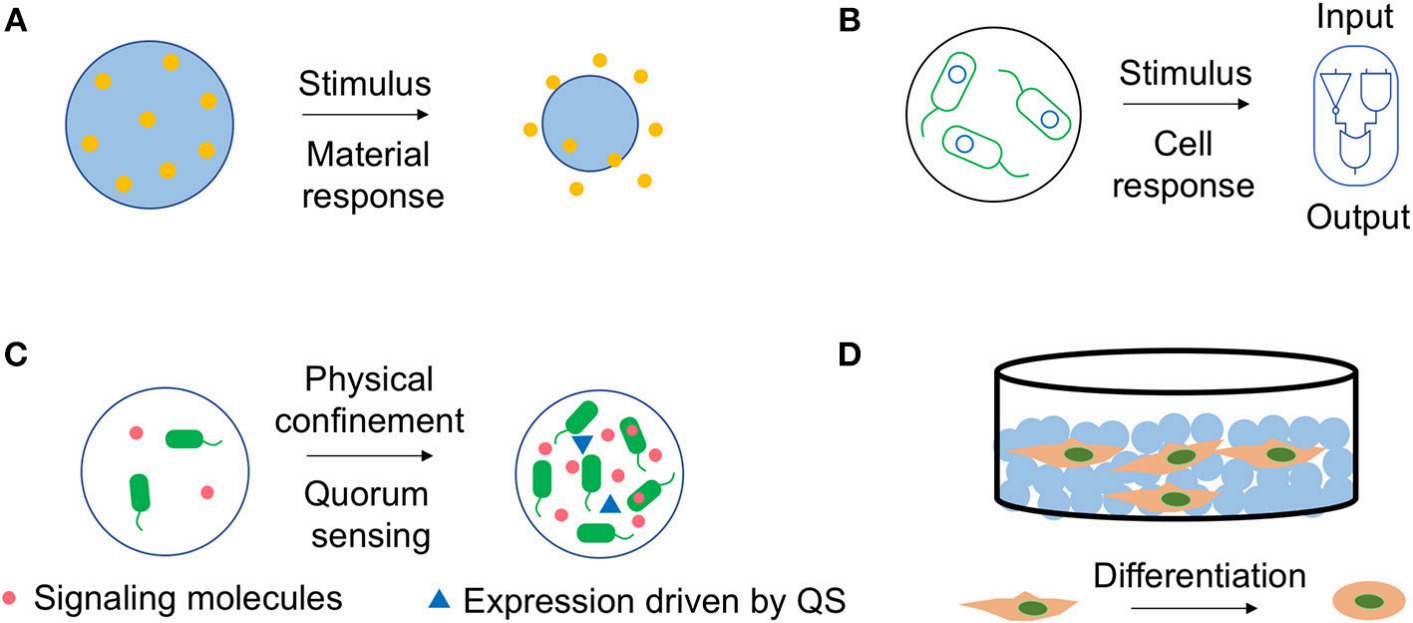
Dynamics inside bio-hybrid systems. **(A)** Materials can respond to external stimulus and release the cargo. Environmental stimulus, such as pH or temperature can trigger the swelling to shrinking transition of the nano- or microgels, and effectively deliver the cargo. **(B)** Engineered cells can respond to external signals and express the functional proteins. Engineered cells encapsulated in polymeric microgels can sense certain signals, such as small molecules and respond accordingly due to the synthetic gene circuit (blue circle). The polymeric capsules trap the cells, but allow the free diffusion of the signaling molecules and functional proteins expressed by cells. **(C)** Cells carrying quorum sensing (QS) circuit can sense their density inside microgels. Quorum sensing bacteria produce and release chemical signaling molecules called autoinducers (red dot), which accumulate in concentration as a function of cell density and regulate gene transcription (blue triangle) as a response. The hybrid of microgels and cells provides a man-made system that exhibits behavior analogous to QS. **(D)** Cells can respond to chemical or physical properties of the scaffolds with different morphogenesis, proliferation or differentiation behaviors. Nano- or microgels can be assembled into 2D or 3D scaffolds. The physical or chemical features of the nano- or microgels will affect their interactions with cells and direct morphogenesis, proliferation or differentiation of cells. The blue circle presents the nano- or microgels. The MSCs imbedded in the scaffold made by the microgels are able to differentiate into a different cell type due to the interactions between the cells and materials.

Besides the general requirements for a drug delivery system, such as optimum loading capacity and biocompatibility, effective delivery of drugs at correct time and location is always considered a big challenge. It requires the system to sense the signals (environmental sensitivity) and actively respond to them (controlled release). Nano- or microgels are potential candidates since the materials can be engineered to sense the environmental cues, while the release of the drugs can be triggered by the volume change of materials (Figure 2A), or the pH induced hydrolysis (Liu et al., 2014; Yu et al., 2016).

Responsive nano or micro-drug delivery systems have been extensively studied in cancer therapy. Due to the weak acidic micro-environment of tumors, pH-responsive nano, or microgels carriers with acid-cleavable bond are usually designed to ensure the controlled drug release. Xiong et al. reported a drug carrier, poly(N-isopropylacrylamide-co-acrylic acid) nanogel with dual temperature and pH sensitivity (Xiong et al., 2011). The system precipitated on the heated cancer tissues (~42°C, local hyperthermia) due to the hydrophilic to hydrophobic transition when temperature was higher than its LCST. The anti-cancer drug doxorubicin (DOX) was covalent linked to the nanogel via an acid-cleavable bond, which could be released efficiently in the acidic microenvironment of tumor tissues. The system utilized both the temperature and pH sensitivity of the materials, as well as acid-cleavable design to ensure the controlled release missions.

In another work, Song et al. developed a responsive nanogel system to deliver both a chemotherapeutic drug (paclitaxel) and a cytokine (interleukin-2) to combat tumors (Song et al., 2017). They first modified the chitosan into two oppositely charged chitosan derivatives. The nanogels were then assembled by mixing these two chitosan derivatives, and further photo-crosslinked with addition of hydroxypropyl-β-cyclodextrin acrylate. The pH responsiveness of nanogel to a weak acidic tumor microenvironment could be fine-tuned by adjusting the formulation, while incorporation of hydroxypropyl-β-cyclodextrin acrylate increased the encapsulation efficiency of paclitaxel (very low solubility in aqueous phase). This responsive bio-hybrid nanogel system integrated chemotherapy and immunotherapy, and significantly enhanced the antitumor activity with improved calreticulin exposure and antitumor immunity.

These responsive nano- or microscale biohybrid system are also widely used in controlled diabetic therapeutic delivery. For example, Sung et al. developed a pH-responsive system consisting of chitosan and poly(γ-glutamic acid) for oral insulin delivery (Sung et al., 2012). Poly(γ-glutamic acid) was incorporated since it could conjugate with insulin via Zn^2+^ to enhance the loading efficiency. The acidic environment of gastric medium in the stomach stabilized the system due to the ionization of chitosan backbones. At intestine (pH ~ 7.0–8.0), the chitosan chains were deprotonated and collapsed. The swollen to shrunk transition of the encapsulating materials enabled the insulin release.

The stimulus-responsive features of nano- or microgels can be further coupled with other biologics, such as an enzyme, to achieve a more sophisticated signal-sensing capability. Gu et al. reported an injectable microgels for controlled glucose-responsive release of insulin by integrating a pH-responsive chitosan matrix, enzyme nanocapsules and insulins (Gu et al., 2013). The enzyme converted the glucose into the gluconic acid at the hyperglycemic condition, and therefore swelled the chitosan matrix due to the protonation of the chitosan networks. Consequently, these microgels were self-regulating and able to release insulins at the basal or higher rate based on normal or hyperglycemic conditions.

### Controlled Release Systems—Response From Cells

Not only materials, engineered cells can also respond to the external signals accordingly (Figure 2B). Indeed, cell-based therapies are considered as one of the most promising next generation medicines. Various synthetic gene circuits have been assembled to treat diverse diseases, including metabolic disorders, cancer and immune diseases (Bulter et al., 2004; Higashikuni et al., 2017; Xue et al., 2017).

Wang et al. reported a pain management strategy based on microencapsulated cell-engineering principles (Wang et al., 2018). The engineered cells can respond to volatile spearmint aroma and produce an analgesic peptide (huwentoxin-IV) that selectively inhibits the pain-triggering voltage-gated sodium channel. Engineered cells were encapsulated in alginate capsules (400 μm). Their results showed that mice (chronic inflammatory and neuropathic pain model) implanted with the capsules demonstrated reduced pain-associated behaviors on oral or inhalation-based intake of spearmint essential oil, with negligible cardiovascular, immunogenic, and behavioral side effects.

In another example, Shao et al. demonstrated a smartphone-assisted treatment of diabetes in mice by microgel encapsulated cells (Shao et al., 2017). In their design, the implanted capsules carried both optogenetically engineered cells and wirelessly powered far-red light (FRL) LEDs (light-emitting diodes). The far-red light LEDs were remotely controlled by smartphone programs or bluetooth-active glucometer in a glucose-dependent manner. Optogenetically engineered cells could respond to FRL based on the bacterial light-activated cyclic diguanylate monophosphate (c-di-GMP) synthase, and activate the expression of mouse insulin. The mouse insulin would then diffuse out from the capsules to control the glucose level in blood.

### Physical Confinement—Response From Engineered Bacterium by Quorum Sensing

Quorum sensing (QS) refers to the ability of organisms to detect and respond to the population density with a specific behavior (Figure 2C; Scott et al., 2017; Shuma and Balazs, 2017). QS plays an essential role in the life cycle of bacteria, yeast, as well as social insects. Designing a man-made system mimicking QS behavior, that is sensing and responding to the system size and density is important but challenging (Ford and Silver, 2015; Li et al., 2017; Shuma and Balazs, 2017). In this notion, polymeric microgels can be integrated with QS cells, since the microgels provide a natural spatial segregation in differentiating the interior and exterior environments. Therefore, the cell populations in individual microgel are effectively insulated from the surrounding environment and other microgels. The porous structure traps the cells, but allows the free diffusion of nutrients and signaling molecules.

In this notion, Huang and Lee demonstrated an engineering safeguard to prevent unintended proliferation by coupling collective survival and environment sensing of bacteria (Lopatkin et al., 2016). Programmed by an engineered circuit, the cells can produce beta-lactamase (BlaM), which is able to degrade beta-lactam antibiotics, such as carbenicillin (Cb) only at a sufficiently high density regulated by QS. At the same time, sufficient cells were required to produce and secrete enough BlaM for population survival. Consequently, the cells inside the microgel can sense the physical confinement, produce BlaM and get rescued due to their high densities. Those escaping from microgel, instead, will be eliminated.

### Interactions With Cells as Scaffold—Response From Cells

Nano- or microgels have been used as building blocks for scaffolds due to its several advantages including ease of fabrication and rapid response to stimulus (Gan et al., 2009; Dhandayuthapani et al., 2011). Cell adhesion on two-dimensional surface or three-dimensional scaffold has been a focus of biophysical research (Dai and Ngai, 2013). Multiple physical or chemical features of the nano- or microgels can dictate their interactions with cells, including the composition, physical properties (porosity, stiffness, etc.) and topography (Leach and Whitehead, 2018). These interactions will direct cells morphogenesis, proliferation, or even differentiation.

Mesenchymal stem cells (MSCs) have been a focus in cell-based therapies for tissue repair and regeneration because of their multilineage differentiation potentials (Augello et al., 2010; Boeuf and Richter, 2010). Growth factors and other chemical inductive cues can effectively induce MSCs differentiation. However, this approach suffers from its own restrictions including the potential off-target effects when treated in large dosages, and long-term maintenance of phenotype after the removal of these cues (Leach and Whitehead, 2018). It has been proven that extracellular matrix (ECM) can direct the MSCs fate through the physical interactions (Engler et al., 2006). Multiple studies have used nano- or microgels as building blocks to assemble the extracellular matrix (ECM) and investigate their effects on the MSCs differentiation (Figure 2D).

From composition perspective, Dai et al. demonstrated that the interactions between MSCs and pNIPAM-AA (poly-N-isopropylacrylamide-acrylic acid) microgels can have a direct effect on osteogenesis of MSCs. Due to the existence of carboxyl groups in the microgels, supplementing microgels could either absorb the free calcium ions and prevent the osteogenesis (supplementing during early osteogenesis), or bind the calcium deposited cells (supplementing during the late osteogenesis) to further promote the osteogenesis through its interactions with cells.

In another work, Caldwell et al. assembled the PEG (polyethylene glycol) microgels into a microporous, covalently linked material, and seeded the human mesenchymal stem cells (hMSCs) into the porous scaffold (Caldwell et al., 2017). To assemble the scaffold, two PEG microgels functionalized with DBCO (dibenzocylcooctyne) or azide groups were separately prepared, and then mixed for crosslinking. Especially, they used two conditions (low shear and high shear) to generate microgels with different sizes. Microgels formed using low shear (votex) had a mean particle diameter of 120 μm (PEG microgels with DBCO) and 130 μm (PEG microgels with azide). Microgels formed using high shear (sonication) were an order of magnitude smaller, with average particle sizes of 16 μm (PEG microgels with DBCO), and 15 μm (PEG microgels with azide). While cells showed high survival rate in both cases, their morphology differed significantly in two scaffolds made by small or large microgels. The cells seeded in the networks made of small microgels had only small protrusions into the matrix, with only diffuse actin fibers present. Comparatively, in the networks made by the big microgels, hMSCs spread and exhibited visible actin fibers.

## Conclusions

Nano- or microgels with tunable properties and sensitivities to external stimulus, have served not only as ideal model systems to explore fundamental questions in physical science, but also as suitable matrixes for diverse applications. With the fast growth in the area of synthetic biology, the integration of engineered microorganisms and polymeric carriers open another door to address both the fundamental problems in population dynamics and evolution, and the real challenges in biotechnology and medicines. This brief review covers several commonly used natural and synthetic nano- or microgels systems, and some of their applications when integrated with diverse biologics spanning from protein drugs, microbiome to mammalian cells. Especially, the dynamics inside the integrated systems to realize the desired function, either from the side of materials or the living organisms, are highlighted.

It is noteworthy that for each nano- or microgels system we are discussing here, it still bears some intrinsic limitations. For example, the typical method for alginate polymerization is introducing divalent cations, such as Ca^2+^ or Ba^2+^, normally at a relatively high concentration comparing to the intracellular conditions. That means, the introduced ions may shift the physiological status of living organisms, complicating the biological processes. For many synthetic polymer system (e.g., pNIPAM), polymerization and crosslinking are mostly initiated by the radical ions. However, either chemical or light-induced method is detrimental to living organisms due to the strong oxidizing ability. Therefore, it is difficult to encapsulate the living cells inside these systems.

Although we have discussed various modes of dynamics inside the bio-hybrid systems, it is noted that all these dynamics remain in one-direction, from the side of either materials, or cells. We hardly find two-directional communications between the materials and biologics in a published bio-hybrid system, which may possess enormous potentials in both fundamental studies and real applications. For example, the cells can respond to physical confinement from the materials, while the materials can probe the dynamics of the cells. In this notion, we are expecting to design and discover bio-hybrid nano- or microgels systems with more delicate and sophisticated functional dynamics in the future.

## Author Contributions

ZD conceived and wrote the manuscript. SH wrote the manuscript. All authors listed have made a substantial, direct and intellectual contribution to the work, and approved it for publication.

### Conflict of Interest Statement

The authors declare that the research was conducted in the absence of any commercial or financial relationships that could be construed as a potential conflict of interest.
